# Optimising Management of Patients with Heart Failure with Preserved Ejection Fraction in Primary Care (OPTIMISE-HFpEF): rationale and protocol for a multi-method study

**DOI:** 10.3399/bjgpopen19X101675

**Published:** 2019-11-27

**Authors:** Faye Forsyth, Jonathan Mant, Clare J Taylor, FD Richard Hobbs, Carolyn A Chew-Graham, Thomas Blakeman, Emma Sowden, Aaron Long, Muhammad Zakir Hossain, Duncan Edwards, Christi Deaton

**Affiliations:** 1 Senior Research Nurse, Primary Care Unit, Department of Public Health & Primary Care, University of Cambridge, Cambridge, UK; 2 Professor of Primary Care Research, Primary Care Unit, Department of Public Health & Primary Care, University of Cambridge, Cambridge, UK; 3 General Practitioner and NIHR Academic Clinical Lecturer, Nuffield Department of Primary Care Health Sciences, University of Oxford, Oxford, UK; 4 Nuffield Professor of Primary Care Health Sciences, Nuffield Department of Primary Care Health Sciences, University of Oxford, Oxford, UK; 5 Professor of General Practice Research, School of Primary, Community and Social Care, Faculty of Medicine and Health Sciences, Keele University, Keele, UK; 6 Clinical Senior Lecturer in Primary Care, Centre for Primary Care, Division of Population Health, Health Services Research & Primary Care, University of Manchester, Manchester, UK; 7 Research Associate, Centre for Primary Care, Division of Population Health, Health Services Research & Primary Care, University of Manchester, Manchester, UK; 8 Assistant Trial Manager, Nuffield Department of Primary Care Health Sciences, University of Oxford, Oxford, UK; 9 Research Assistant, Health Services Research, School of Primary, Community and Social Care, Faculty of Medicine and Health Sciences, Keele University, Keele, UK; 10 Senior Clinical Research Associate, Primary Care Unit, Department of Public Health & Primary Care, University of Cambridge, Cambridge, UK; 11 Florence Nightingale Foundation Clinical Professor of Nursing, Primary Care Unit, Department of Public Health & Primary Care, University of Cambridge, Cambridge, UK

**Keywords:** cardiovascular diseases, care of older people, research methods (other), heart failure, aged, general practice, primary health care

## Abstract

**Background:**

Heart failure with preserved ejection fraction (HFpEF) is less well understood than heart failure with reduced ejection fraction (HFrEF), with greater diagnostic difficulty and management uncertainty.

**Aim:**

The primary aim is to develop an optimised programme that is informed by the needs and experiences of people with HFpEF and healthcare providers. This article presents the rationale and protocol for the Optimising Management of Patients with Heart Failure with Preserved Ejection Fraction in Primary Care (OPTIMISE-HFpEF) research programme.

**Design & setting:**

This is a multi-method programme of research conducted in the UK.

**Method:**

OPTIMISE-HFpEF is a multi-site programme of research with three distinct work packages (WPs). WP1 is a systematic review of heart failure disease management programmes (HF-DMPs) tested in patients with HFpEF. WP2 has three components (a, b, c) that enable the characteristics, needs, and experiences of people with HFpEF, their carers, and healthcare providers to be understood. Qualitative enquiry (WP2a) with patients and providers will be conducted in three UK sites exploring patient and provider perspectives, with an additional qualitative component (WP2c) in one site to focus on transitions in care and carer perspectives. A longitudinal cohort study (WP2b), recruiting from four UK sites, will allow patients to be characterised and their illness trajectory observed across 1 year of follow-up. Finally, WP3 will synthesise the findings and conduct work to gain consensus on how best to identify and manage this patient group.

**Results:**

Results from the four work packages will be synthesised to produce a summary of key learning points and possible solutions (optimised programme) which will be presented to a broad spectrum of stakeholders to gain consensus on a way forward.

**Conclusion:**

HFpEF is often described as the greatest unmet need in cardiology. The OPTIMISE-HFpEF programme aims to address this need in primary care, which is arguably the most appropriate setting for managing HFpEF.

## How this fits in

HFpEF is poorly recognised and managed in the community. As HFpEF is set to become the dominant form of heart failure (HF), it is vital that effective primary care-based programmes of identification and management are developed. The OPTIMISE-HFpEF programme of research aims to fill this void.

## Introduction

HFpEF is less well understood than HFrEF and is associated with greater diagnostic difficulty and management uncertainty.^1^ Half of all HF cases may be attributable to HFpEF, and prevalence is rising at a rate of 1% annually.^1–3^ Although mortality for all-cause HF in the UK has modestly improved, no treatment has yet been shown to improve mortality and morbidity in HFpEF.^4,5^ Lack of evidence for pathophysiological mechanisms underpinning the disease, effective pharmacotherapies, and disease management programmes specifically targeting HFpEF hamper progress. It is perhaps unsurprising then that clinicians may, to coin Oktay and Shah, *'* *approach HFpEF with diagnostic and therapeutic nihilism* *'*.^6^

This article presents the rationale and protocol for the OPTIMISE-HFpEF research programme (https://www.optimisehfpef.phpc.cam.ac.uk/), in line with the Strengthening the Reporting of Observational Studies in Epidemiology (STROBE) statement.^7^ This project aims to explore the views of people with HFpEF and the multiple stakeholders involved in HFpEF care; phenotype a UK cohort; and undertake consensus methods to develop an optimised programme that would provide guidance to clinicians in diagnosing and managing HFpEF.

### Background and rationale

The emergence of HFpEF as a clinical syndrome has been, and continues to be, controversial.^4^ There are uncertainties along the entire spectrum from mechanisms and diagnosis to treatment and management. Understanding of underlying pathophysiological processes continues to develop through research, and varying models have been proposed to explain the abnormal cardiac structure and function observed.^2,4,8^ The current preferred paradigm is that microvascular endothelial inflammation, driven by coexisting conditions, leads to myocardial inflammation and fibrosis that in turn results in increased oxidative stress and alterations in cardiomyocyte signalling.^2,8^ Diagnostically, while definitions and criteria have been tightened, universal consensus and a definitive algorithm are lacking.^9–11^ In treatment terms, evidence-based pharmacological therapies and device options are limited and developments lag behind those observed in HFrEF.^4^ Disease management programmes consistently demonstrating reduced mortality and re-hospitalisation rates in HF do not frequently include or explicitly analyse data on HFpEF;^12,13^ therefore, benefits observed cannot confidently be extrapolated. Lastly, a persistent gap between best and current practice exists, which is compounded by a lack of integrated care.^14–16^

The outcome of these challenges in HFpEF is high rates of hospitalisation, mortality, poor functioning, and low quality of life.^3,17^ Some authorities have already called for dedicated HFpEF clinical programmes, and lessons will be garnered from pioneering initiatives.^18^ However, while they acknowledge the necessity of the primary care provider, there is a lack of specificity and focus on the role of general practice.

General practice has a key role in all parts of the HF patient pathway, from initiating diagnosis to long-term management;^19,20^ a pathway enshrined in national guidelines.^21,22^ However, even national guidelines pay sparse attention to HFpEF, dedicating the majority of the content to HFrEF (see Appendix A in supplementary materials for an overview of current recommendations). They do not address or troubleshoot many of the key issues debated in HFpEF research; for example, the lack of specificity of echocardiogram to accurately capture diastolic left ventricle dysfunction,^23–25^ the frequent absence of echocardiographic evidence of structural heart disease in invasively confirmed HFpEF,^26^ and the poor sensitivity of natriuretic peptides in HFpEF.^27,28^ All the latter are key components of HFpEF diagnostic algorithms in the UK. Moreover, they lack the necessary focus on exercise, which has pleiotropic effects on systemic endothelial function and therefore improves symptoms and quality of life.^29^

Others have already argued an effective pathway to achieve cohesive primary and/or specialist co-management remains elusive and the current structure of care is suboptimal.^30^ In HFpEF, the need to establish this is ever more pressing given the increasing prevalence, the burden of multimorbidity, and the complex interplay of medical, psychological, behavioural, and social factors for this group.^31^ Optimally managing a vulnerable and complex population of older adults, in a pragmatic and patient-centred way, is arguably better addressed by generalists familiar with patient preferences and priorities and the application of comprehensive and multidimensional assessment.^32^

### Why this research is needed

HFpEF is poorly recognised and managed in the community. As HFpEF is set to become the dominant form of HF, it is vital that effective primary care-based programmes of identification and management are developed. The OPTIMISE-HFpEF programme of research aims to fill this void by: (1) finding out what patients and providers want; (2) understanding what people with HFpEF are like in the UK, through detailed phenotypic characterisation of a large cohort; and (3) exploring common problematic areas like transitions of care (any move from one care setting to the next, most often hospital to home), hospitalisations, and the support of carers (see Table 1). Results generated will be synthesised, and both face-to-face and online consensus methods employed to develop and refine an optimised programme of management.

**Table 1.** Work package details

	**MRC complex intervention stage mapping**
**WP1**	**De** **scription**	Systematic review of disease management programmes tested in HFpEF populations (see Prospero: CRD42017067980).	Identifying the evidence base.
	**Loc** **ation**	Cambridge, UK	
	**Recruitment**	N/A	
	**Data collection**	Commenced October 2017 – completed September 2019	
**WP2a**	**Des** **cription**	Qualitative interview study to determine patient and health professionals’ preferences, perspectives on burden of illness and treatment, care requirements, and organisation of services and/or support in HFpEF.	Identifying and/or developing theory.Modelling process and outcomes.
	**Loc** **ation**	Collaborative, multi-site study involving Cambridge, Keele, and Manchester (UK). Sites are utilising the NIHR Primary Care Research Network to identify general practices in their region through which recruitment of patients and primary care clinicians will be managed. Secondary and primary care-based HFS services will be approached to augment recruitment. Other healthcare providers and commissioners will be identified via local networks.	
	**Rec** **ruitment**	Commenced October 2017 – planned end March 2020	
	**Data collection**	Data collection involves face-to-face or telephone interview with patients ±their carers and healthcare professionals managing or structuring care for patients with HFpEF, including but not limited to HFS nurses, cardiologists, GPs, practice nurses, healthcare commissioners, and rehabilitation specialists.	
**WP2b**	**Des** **cription**	Prospective longitudinal observational cohort study that will identify probable HFpEF patients, confirm HFpEF status, characterise the cohort at baseline, and prospectively follow-up confirmed HFpEF cases for 1 year.	Modelling process and outcomes.Estimate recruitment and retention.Determine sample sizePreliminary testing of procedures.
	**Loc** **ation**	Collaborative, multi-site study involving the Universities of Cambridge and Oxford, and Cardiology/Care of the Elderly services at North West Anglia and Guy’s and St. Thomas’ NHS Foundation Trust. Cambridge and Oxford will utilise the NIHR Primary Care Research Network to identify general practices in their region through which recruitment of patients will be managed.	
	**Rec** **ruitment**	Commenced July 2018 – planned end July 2020.	
	**Data collection**	Data collection involves a baseline visit where a diagnostic echocardiogram will be performed (previously performed diagnostic echocardiograms will be used at secondary care sites) to confirm the presence or absence of HFpEF. Additional assessments at baseline are described in Table 2.	
**WP2c**	**Des** **cription**	Qualitative interview sub-study employing a framework analysis approach to explore hospitalisation in HFpEF patients, transitions of care, and their carers’ perspectives.	Identifying and/or developing theory.Modelling process and outcomes.
	**Loc** **ation**	Sub-study within the Cambridge longitudinal cohort sample.	
	**Rec** **ruitment**	Commenced December 2018 – planned end July 2020.	
	**Data collection**	Data collection involves face-to-face interviews with patients and their carers. Basic demographic information and interview notes and/or reflections will also be collected.	
**WP3**	**Des** **cription**	Summary statement and questions presented to stakeholders to gauge consensus, explore disparity, identify sticking points, and elicit programme refinements.	Identifying and/or developing theory.Modelling process and outcomes.Preliminary testing of procedures.Understanding change process.
	**Location**	UK-wide, diverse sample of non-collocated 'experts' with various levels and domains of expertise (including but not limited to: patients, primary care physicians, cardiologists, echocardiography specialists, HFS, and heart failure charities).	
	**Rec** **ruitment**	Commenced April 2019 – planned end December 2020.	
	**Data collection**	Using a structured online system, the experts will be asked to discuss the summary statement. Comments are aggregated, then quantitatively and qualitatively analysed using statistical modelling techniques to enable decision-making based on the input from the expert panellists.	

PROSPERO is the international prospective register of systematic reviews, accessible at https://www.crd.york.ac.uk/prospero/

HFpEF = heart failure with preserved ejection fraction. HFS = heart failure specialist. MRC = Medical Research Council. N/A = not applicable. NIHR = National Institute for Health Research. WP = work package.

### Aim

The primary aim is to investigate HFpEF with the view to developing an optimised programme that is informed by the needs and experiences of people with HFpEF and healthcare providers. A secondary aim is to gauge stakeholder consensus on the optimised programme using online and face-to-face consensus methods.

## Method

### Study design

**Figure 1.**
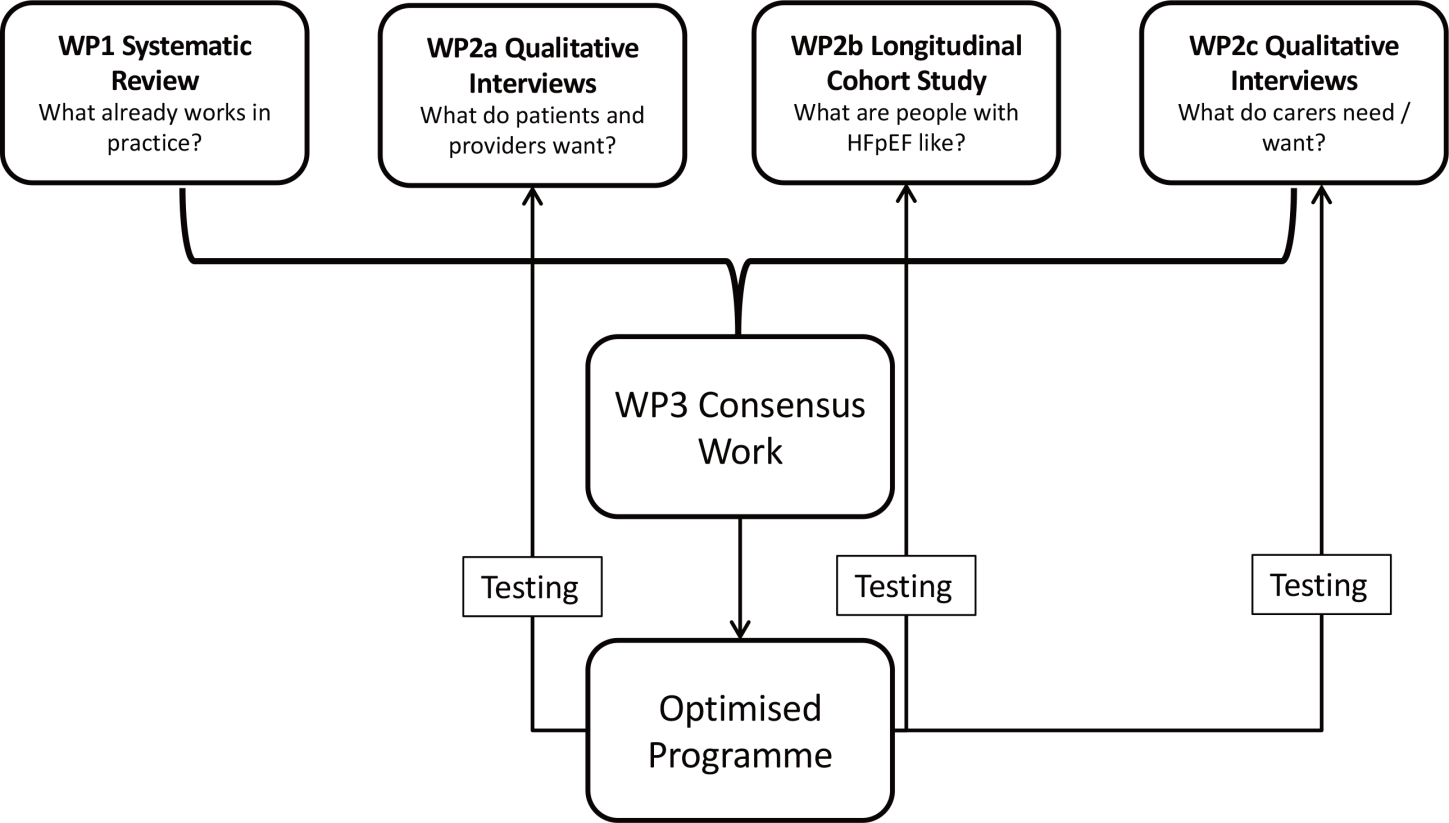
OPTIMISE-HFpEF programme of research HFpEF = heart failure with preserved ejection fraction. WP = work package.

An optimised programme would constitute a complex intervention; therefore, the multi-stage cyclical process consisting of four key elements outlined by the Medical Research Council (MRC) has been adopted.^33^ The resulting mixed-methods approach consisting of five WPs is cognisant of these key elements (see Table 1 and Figure 1) and has been designed to maximise opportunities for theorising, iterative development, and pragmatic modelling of contextual, organisational, and logistical factors. Each of the WPs also has ‘legacy elements’; therefore, the emerging programme of care can be tested and critiqued by ‘end users’, either qualitatively via networks and cohorts of patients, carers, and providers (WP2a and WP2c), or quantitatively in an established cohort of HFpEF patients (WP2b).

### Setting

Multiple sites were involved to enhance the diversity of the sample in each cohort (see Table 1).

### Participants

Participants in WP2a and WP2b must meet the following inclusion criteria: adult patients with diagnosed or suspected HFpEF, and the ability to communicate in English. Diagnosed or suspected HFpEF was defined as anyone diagnosed with non-valvular HF that (i) were not diagnosed with left ventricular systolic dysfunction or have a documented ejection fraction (EF) <50%; or (ii) have a reported ‘normal’ or preserved EF (that is, >50%); or (iii) have an echocardiogram reporting structural heart disease or diastolic dysfunction without moderate to severe systolic dysfunction. Although the European Society of Cardiology guideline is used to diagnose HFpEF^9^ in WP2b, this pragmatic definition was employed given the literature detailing the challenges of identifying patients with HF from primary care records.^34–36^

The following exclusion criteria were applied to WP2a and WP2b: any severe neurocognitive condition that would confound outcome assessment; New York Heart Association class IV classification; or other life-threatening condition. Heart failure exacerbations resulting in hospitalisation in the 6 weeks before screening were included in WP2b owing to the nature of the physical assessments. Participants in WP2c will include patients from WP2b who have a confirmed diagnosis of HFpEF and have experienced hospitalisation for HF or a comorbid condition. Adult carers will be invited via patient participants. No specific inclusion or exclusion criteria will be applied to WP3, with the aim of recruiting a heterogeneous panel representative of a range of views.

**Figure 2.**
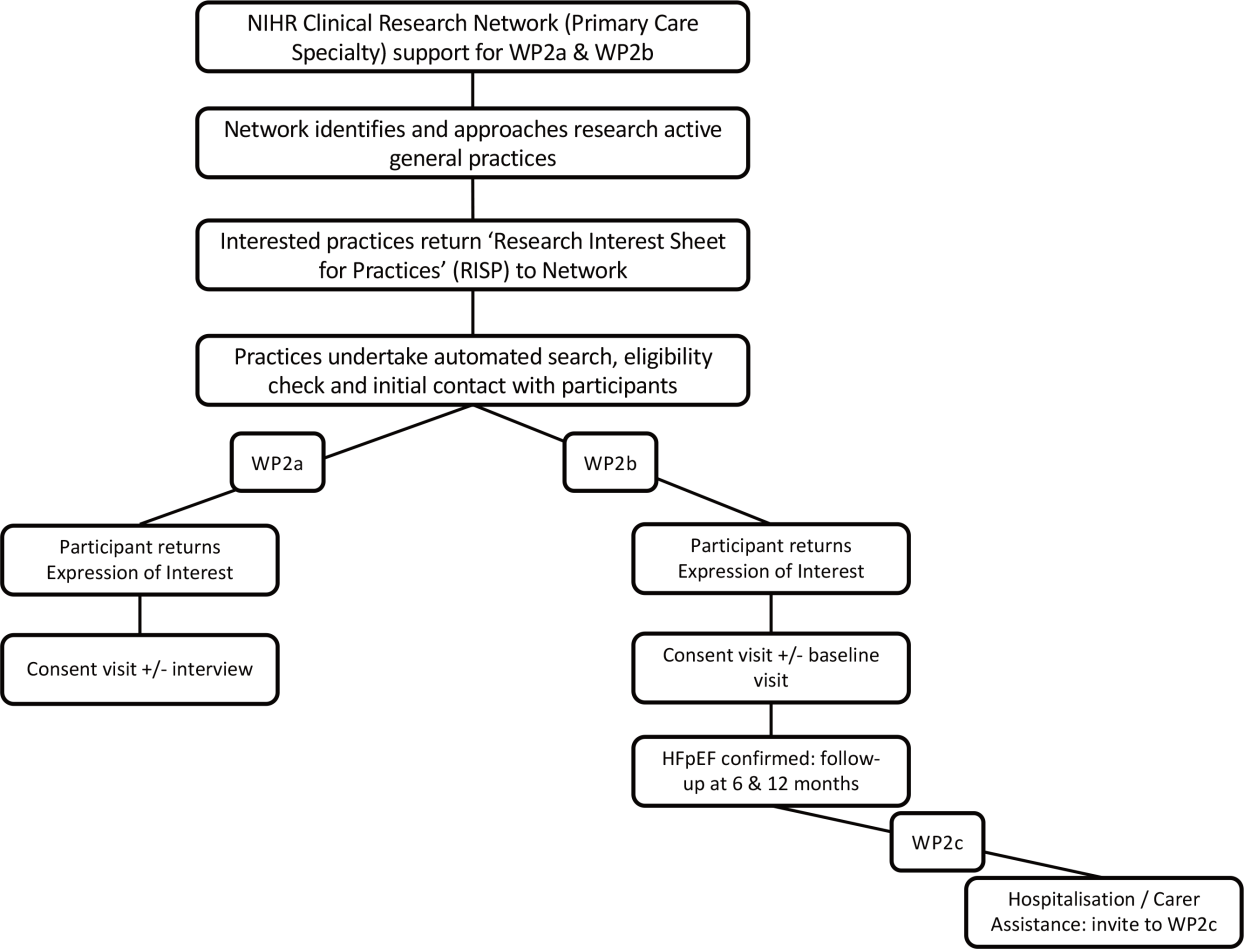
Flow of participants HFpEF = heart failure with preserved ejection fraction. WP = work package.

Patients with HFpEF in WP2a and WP2b will be identified with the support of the National Institute for Health Research Clinical Research Networks (NIHR CRN). In brief, the NIHR CRN offers a range of support to help researchers plan, set up, and deliver research. In this case, assistance was sought to gain access to general practices that were provided with an electronic medical record screening algorithm to enable identification of potential participants. A GP within the practice screened against the inclusion and exclusion criteria before inviting eligible patients. Patients interested in taking part were asked to return an expression of interest form to the research team. The research team contacted those interested to discuss the study in detail (including any risks and/or benefits, and right to withdraw) before written informed consent was sought (see Figure 2). Purposive sampling will be used in WP2c to target patients with confirmed HFpEF that have experienced a hospitalisation; snowball sampling will be used to recruit their carers. The WP3 sample will be recruited via current WPs and local networks.

A patient advisory group is active within the study, and continues to provide input.

### Variables and data sources

WP1 was a systematic review of controlled studies of HF-DMPs that included patients with HFpEF. Four bibliographic databases were searched, yielding 6089 titles that were screened against an agreed protocol (see PROSPERO https://www.crd.york.ac.uk/prospero/). Eighteen studies met the inclusion criteria, representing 1866 patients with HFpEF. All studies were assessed against a ‘disease management programme taxonomy’ and scored for complexity, intensity, and bias. The review concluded HF-DMPs may improve mortality, hospitalisation rates, self-care, and quality of life in patients with HFpEF; however, further research specifically tailored to HFpEF is required.

In WP2a and WP2c, sociodemographic data, interview notes and/or reflections, and interview recordings will form the main data sources. In WP2c, the caring relationship and arrangement will be documented and the Carer Support Needs Assessment Tool (CSNAT) completed.^37^ Hospitalisations will be validated via Hospital Episode Statistics (details of all admissions, accident and emergency attendances, and outpatient appointments at NHS hospitals in England).

WP2b will collect sociodemographic, behavioural, clinical, and quality-of-life data at baseline, 6, and 12 months (Table 2). The aim is to phenotype and follow a UK cohort over a 1-year period. As such, there is no primary outcome or comparison; analysis will be descriptive and exploratory. To date, much of the insight into the clinical characteristics of HFpEF comes from randomised controlled trials of drug therapies. Such studies have stringent inclusion criteria that exclude the very old and those with significant multimorbidity or functional impairments — arguably the ‘typical’ patient with HFpEF.^20^ Refined characterisation of a representative, real-world cohort will help with effective targeting of the components in the final programme of care.

**Table 2.** WP2b clinical and behavioural variables

**Parameter**	**Measure**	**Description**
**Physical characteristics**	Anthrop	Height in centimetres, weight in kilograms, BMI kg/m^2^
	Vitals	Blood pressure (mmHg)
		Respiratory rate (breaths per minute)
		Pulse rate (beats per minute)
**Past medical history**	N/A	Past and current medical problems and medications will be extracted from primary and secondary care records
**Clinical events**	HES	Hospital Episode Statistics (date, length of time and index reason for hospitalisations, accident and emergency attendances, and outpatient appointments)
**Heart function**	ECG	12-lead ECG
	Echo	Detailed echocardiogram with high-quality diastolic and right ventricle functional assessment
**Arterial stiffness** ^a^	PWV	A validated reproducible technique to investigate the clinical relevance of vascular and arterial stiffness^36^
**Peripheral oedema**	**N/A**	Clinical assessment of oedema including level (extent) and presence or absence of pitting
**Breathlessness and fatigue**	mBORG	Valid, reliable measure of the intensity of the sensation of breathlessness and fatigue^37^
**Frailty**	CFS	A validated measure of frailty based on clinical judgment^38^
	SHARE-FI	A validated automated instrument that generates a pre-calculated, population-representative, and sex-specific frailty class^39^
	eFI	eFI uses routine medical record data to identify older people with mild, moderate, and severe frailty and will be abstracted from primary care records^40^
**Comorbidity**	CCI	Widely used validated measure of 1-year mortality risk and burden of disease^41^
**Cognition**	MoCA	The MoCA is a brief cognitive screening tool with high sensitivity and speciﬁcity for detecting mild cognitive impairment^42^
**Physical functioning and activity**	6MWT	A standardised submaximal test of aerobic capacity, validated in multiple populations and conditions^43^
	GS	Gait speed measured over 10 metres, a valid objective measure of functional mobility^44^
	Acceler	Objective measure of activity obtained via Axivity AX3 wrist-worn triaxial accelerometer programmed to start at 19:00 hours on the day of the baseline visit (to prevent capturing of protocol forced activity) and capture triaxial acceleration data over a 7-day period at 100 Hz with a dynamic range of +–8 g^45^
**Laboratory testing**	Biochem	Serum sodium, potassium, creatinine, urea, estimated GFR, random plasma glucose
	Haem	White and red blood cell count, haemoglobin, haematocrit, mean cell volume, mean cell haemoglobin, red cell distribution width, platelet count, mean platelet volume, neutrophil, lymphocyte, monocyte, eosinophil, basophil count
**Biomarkers**	HbA1c	An indicator of the average blood glucose concentrations over the preceding ~2 months.
	NP	Natriuretic peptides (NT-proBNP), a diagnostic marker in patients with heart failure
**Dietary intake** ^a^	Interview	One 24-hour dietary recalled will be collected to ascertain over or undernutrition in HFpEF patients
**Anxiety and depression**	HADS	HADS is a widely used questionnaire that screens for the separate dimensions of anxiety and depression and has been validated in multiple populations^32^
**HF QoL**	KCCQ	KCCQ is a valid, reliable and responsive health status measure for patients with chronic heart failure that has been shown to have clinically meaningful changes^33^
**HF self-care**	EHFScBQ	A valid, reliable and practical scale to measure the self-reported self-care behaviour of heart failure patients.^34^
**HF symptoms**	SSQ-HF	Valid and reliable score to assess physical symptoms in patients with heart failure.^35^
**Health-related QoL**	EQ-5D	The EQ-5D is a widely used five-domain patient-based generic questionnaire for self-perceived health assessment. It describes health-related quality of life and has been extensively validated.

6MWT = 6-minute walk test. Acceler = accelerometry. Anthrop = anthropometry. Biochem = biochemistry. BMI = body mass index. CCI = Charlson comorbidity index. CFS = clinical frailty scale. ECG = electrocardiogram. Echo = echocardiogram. eFI = electronic frailty index. eGFR = estimated glomular filtration rate. EHFScBQ = European heart failure self-care behaviours questionnaire. EQ-5D = the EuroQol 5D questionnaire. GS = gait speed. HADS = hospital anxiety and depression score. Haem = haematology. HbA1c = glycated haemoglobin A1c. HES = hospital episode statistics. KCCQ = Kansas City cardiomyopathy questionnaire. mBORG = modified BORG. MoCA = Montreal cognitive assessment. N/A = not applicable. NP = natriuretic peptides. PWV = pulse wave velocity. QoL = quality of life. SHARE-FI = SHARE frailty instrument. SSQ-HF = symptom status questionnaire — heart failure.

^a^Indicates single site sub-study.

The results of findings from all the WPs will be integrated to produce a position article that will form the basis of the questions presented in the modified Delphi online elicitation approach. This methodology produces ratings and rankings of statements as well as qualitative online discussion data from expert panel members.

### Bias and fidelity

All study procedures will be conducted in accordance with the respective protocols (see https://www.optimisehfpef.phpc.cam.ac.uk/) and in line with the principles outlined in the International Conference on Harmonisation Good Clinical Practice guideline.^38^ WP2a and WP2c will use a semi-structured interview template that will guide the interview process while also allowing flexibility for exploration of specific issues. WP2b has a comprehensive Manual of Operations and Procedures to standardise practice in relation to outcome measure collection.

### Study size

WP2a will recruit up to 100 patients and healthcare professionals. WP2c will recruit 40 previously hospitalised patients with HFpEF and their carers. The sample size for the qualitative components is an approximation of the number of subjects required based on previous research; the adequacy will be evaluated during the research process.^39^

The planned sample size in WP2b is 270 patients. This estimate is based on exemplar calculations to ensure confidence in the estimates of characteristics and changes in measures over 1 year, but is also cognisant of practical logistical considerations and rapidly changing diagnostic criteria in HFpEF. Previous research has demonstrated difficulty in confirming HFpEF from primary care records owing to the absence of diagnostic information.^30^ Based on this, it is estimated up to around 25% of referred patients will not have HFpEF after cardiologist review of baseline data. The final sample size was not adjusted for attrition.

### Statistical and qualitative analysis methods

The main data source in WP2a and WP2c will be qualitative semi-structured interview recordings and verbatim transcripts. These will be analysed using the framework method^40^ to gain insight into the perspectives of patients receiving HFpEF care and their carers, and of primary and secondary healthcare providers planning and delivering that care across three regions in England. The framework method employs a structured approach and the main output — a matrix of charted cases, codes, and summarised data excerpts — enables researchers to ‘systematically reduce’ the data to aid interpretation.^40,41^ Data will be organised in NVivo (version 12) and Microsoft Excel (version 14.0.7237.5000) software. A thematic framework (coding scheme) will be developed, before indexing, charting, describing, and interpreting the data.^40,41^ The analysis will be both inductive (data-driven) and deductive (theory-driven), with burden of treatment and minimally disruptive medicine models employed as sensitising frameworks.^42,43^ WP2c will follow a similar analysis pathway; deductive analysis will be driven by burden of treatment theory (hospitalised patients) and the CSNAT (carers).

Where possible, descriptive statistics (frequencies and proportions, mean and standard deviation, or median and interquartile range, as appropriate) will be presented for all WPs. In WP2b, pre-specified baseline comparisons will determine differences by sex, body mass, and presence of frailty. In addition to robust clinical information, the cohort will be described according to patient-reported measures on symptoms, self-management, HF specific quality of life, and physical activity. Reported physical activity will be validated against information from activity monitors regarding both level of activity and time spent sedentary. A similar analysis will be conducted with data from the 6- and 12-month follow-up to determine changes in variables from baseline. Data on outcomes (all-cause and cardiovascular hospitalisations and mortality, length of stay in hospital, readmissions, and timeframes of readmissions) will be collected over the 12-month period.

Analysis in WP3 will be managed via an online platform, which enables quantitative, categorical, and text data analysis from all rounds of the elicitation and is conducted in four stages: preliminary data analysis, analysis of agreement, data modelling, and analysis of changes in responses.^44,45^

### Dissemination

Dissemination of the study findings will include at least one peer-reviewed article from each WP, and presentations at appropriate primary care and specialist conferences.

## Discussion

### Summary

This multi-method programme aims to explore the views of the multiple parties involved in HFpEF care; to characterise a UK cohort of HFpEF patients; and to explore hospitalisation, transitions of care, and carer burden with a view to informing the development of an optimised programme of care. The optimised programme would be developed using consensus methods via an online platform that would refine programme components and preliminarily assess feasibility. The final programme would then undergo further testing in a cohort of patients with confirmed HFpEF.

### Strengths and limitations

The strengths of this programme are the patient, carer, and health professional focus and the particular emphasis on pragmatic, primary care-based solutions. Heart failure is usually identified and managed long term in the primary care setting, however there is an absence of much needed guidance and support available for GPs. The main limitation of this project relates to testing the findings of the consensus work (WP3); this would be subject to future funding.

### Comparison with existing literature

There is a growing body of research in HFpEF owing to its status as the 'greatest unmet need in cardiology’.^29^ However, much of this research is in the highly specialist or tertiary setting, and there is a dearth of literature on the role of generalists in HFpEF identification, diagnosis, and management. Smeets and colleagues have proposed a multi-component optimisation intervention in the primary care setting (OSCAR-HF pilot study); this, however is in HF generally as opposed to HFpEF specifically.^46^

### Implications for research and practice

HFpEF is an underrecognised condition that poses many challenges for clinicians. This programme seeks to understand these problems from the perspective of patients, their carers, and the healthcare professionals who look after them, with a view to establishing an optimised programme of management that is cognisant of these issues.

## Trial registration number

WP1 is registered on PROSPERO (reference number: CRD42017067980). WP2b is registered on ClinicalTrials.gov (reference number: NCT03617848).
